# Neurogenic ST-segment elevation following acute ischemic stroke with hemorrhagic transformation: a case report

**DOI:** 10.1097/RC9.0000000000000898

**Published:** 2026-08-24

**Authors:** Osman Farah Dahir, Abdiwahid Ahmed Ibrahim, Ismail Gedi Ibrahim, Farah Abdullahi Ismail, Mohamed Sheikh Hassan, Said Abdirahman Ahmed

**Affiliations:** a Cardiology Department Mogadishu Somali-Turkish Training and Research Hospital, Mogadishu, Somalia; b Neurology Department, Mogadishu Somali-Turkish Training and Research Hospital, Mogadishu, Somalia; c Interventional Radiology Department, Mogadishu Somali-Turkish Training and Research Hospital, Mogadishu, Somalia; d Department of Pediatrics, Mogadishu Somali-Turkish Training and Research Hospital, Mogadishu, Somalia; e Faculty of Medicine and Surgery, Salaam University, Mogadishu, Somalia

**Keywords:** acute ischemic stroke with hemorrhagic transformation, case report, conservative management, electrocardiography, neurogenic ST-segment elevation, stress cardiomyopathy, stroke–heart syndrome

## Abstract

**Background:**

Neurogenic ST-segment elevation is a rare but clinically significant cardiac manifestation that can occur after acute ischemic stroke with hemorrhagic transformation. It may closely mimic acute ST-elevation myocardial infarction (STEMI), leading to potentially inappropriate diagnostic or therapeutic interventions.

**Case presentation:**

We report on a 46-year-old man who presented with right-sided weakness and expressive aphasia for 1 day. He had a history of untreated hypertension. On admission, vital signs were notable for a BP of 190/100 mmHg and an HR of 100 bpm. The ECG revealed ST-segment elevation in leads V1–V4. Brain MRI confirmed an acute left parietotemporal ischemic stroke with hemorrhagic transformation. Echocardiography showed preserved left ventricular function with mild hypertrophy. Laboratory tests revealed elevated troponin and BNP levels. Given stable hemodynamics and the absence of regional wall motion abnormalities, cardiology recommended conservative management with close monitoring.

**Management and outcome:**

The patient received antihypertensive therapy, mannitol infusion, head-of-bed elevation, and physiotherapy. Serial ECG monitoring demonstrated resolution of ST-segment elevation by day 5. Neurological improvement was progressive, and the patient was discharged after 8 days with follow-up recommendations.

**Discussion:**

This case highlights a reversible pattern of ST-segment elevation secondary to neurogenic myocardial injury following acute ischemic stroke with hemorrhagic transformation. Recognition of this phenomenon is essential to avoid misdiagnosis and unnecessary cardiac interventions.

**Conclusion:**

In patients with acute ischemic stroke and hemorrhagic transformation presenting with ECG changes suggestive of STEMI, integrated neurological and cardiac assessment with serial ECG and biomarker monitoring is crucial to guide safe management and prevent iatrogenic harm.

## Introduction

Acute ischemic stroke with hemorrhagic transformation is a severe neurological event associated with high morbidity and mortality, and it frequently results in systemic complications beyond the primary brain injury. Cardiovascular manifestations, particularly electrocardiographic abnormalities, are common in this context and can closely mimic acute coronary syndromes, such as ST-elevation myocardial infarction (STEMI). Among these manifestations, neurogenic ST-segment elevation is clinically significant because it may trigger invasive cardiac interventions that could worsen neurological outcomes^[^1,2^]^.

The concept of the brain–heart axis describes the complex interplay between the central nervous system and cardiac function. Neurological injuries can disrupt autonomic regulation, particularly when the insular cortex is involved, a region critical for sympathetic and parasympathetic balance^[^3,4^]^.

Acute cerebral injury may precipitate a catecholamine surge, leading to excessive sympathetic activation, coronary microvascular spasm, and myocardial stunning. These changes can result in stress-induced (Takotsubo) cardiomyopathy, characterized by transient left ventricular dysfunction and ECG changes, often without obstructive coronary artery disease^[^3,5,6^]^.

Understanding this neurocardiac connection is essential, as the resulting ECG abnormalities and cardiac biomarker elevations may be misinterpreted as primary cardiac pathology. Differentiating neurogenic myocardial injury from acute coronary events is crucial for patient safety, guiding appropriate conservative management and avoiding potentially harmful antithrombotic therapy^[^4,7^]^.

A case of neurogenic ST-segment elevation in a patient with acute ischemic stroke with hemorrhagic transformation is presented, highlighting the importance of an integrated neurological and cardiac assessment to guide clinical decisions.

## Methods

This case report has been prepared and reported in accordance with the 2025 Surgical Case Report (SCARE) criteria.

## Case presentation

A 46-year-old male patient presented to the emergency department with sudden right-sided weakness and expressive aphasia of 1 day’s duration. He had a history of untreated hypertension, no prior cardiac disease, and no known family history of stroke, cardiovascular disease, or sudden cardiac death. There was no history of chest pain, palpitations, syncope, headache, visual disturbances, or recent trauma. The patient was socially independent and able to perform daily activities without assistance.

### Vital signs and examination

On admission, BP was 190/100 mmHg, HR 100 bpm, oxygen saturation was 100% on room air, and temperature was 36°C. Neurological examination revealed expressive aphasia and right hemiparesis (3/5). Other systemic examinations were normal.

### Timeline of clinical events

Key clinical events are shown in Table 1.

**Table 1:** Timeline of clinical events.

Day	Event
Day −1	Onset of right-sided weakness and expressive aphasia
Day 0	Admission: BP 190/100 mmHg, HR 100 bpm, O_2_ sat 100%, Temp 36°C; neurological exam: right hemiparesis (3/5), expressive aphasia
Day 0	ECG: ST-segment elevation in leads V1–V4 (Fig. 1)
Day 0	Brain MRI: acute left parietotemporal ischemic stroke with hemorrhagic transformation (Fig. 2)
Day 0	Echocardiography: LVEF 60–65%, mild LVH, no regional wall motion abnormalities
Days 1–5	Management: IV antihypertensive therapy, mannitol, head-of-bed elevation, supportive care, and physiotherapy
Days 1–5	Serial troponin: day 1, 0.20 ng/mL; day 2, 0.15 ng/mL; day 3, 0.12; day 4, 0.10 ng/mL; day 5, 0.08 ng/mL; BNP: 215 pg/mL on admission
Day 5	ECG: Resolution of ST-segment elevation (Fig. 3).
Day 6–8	Neurological improvement and continued inpatient physiotherapy
Day 8	Discharge with outpatient follow-up and rehabilitation recommendations

LVEF, left ventricular ejection fraction.

### Diagnostic assessment

**Figure 1.**
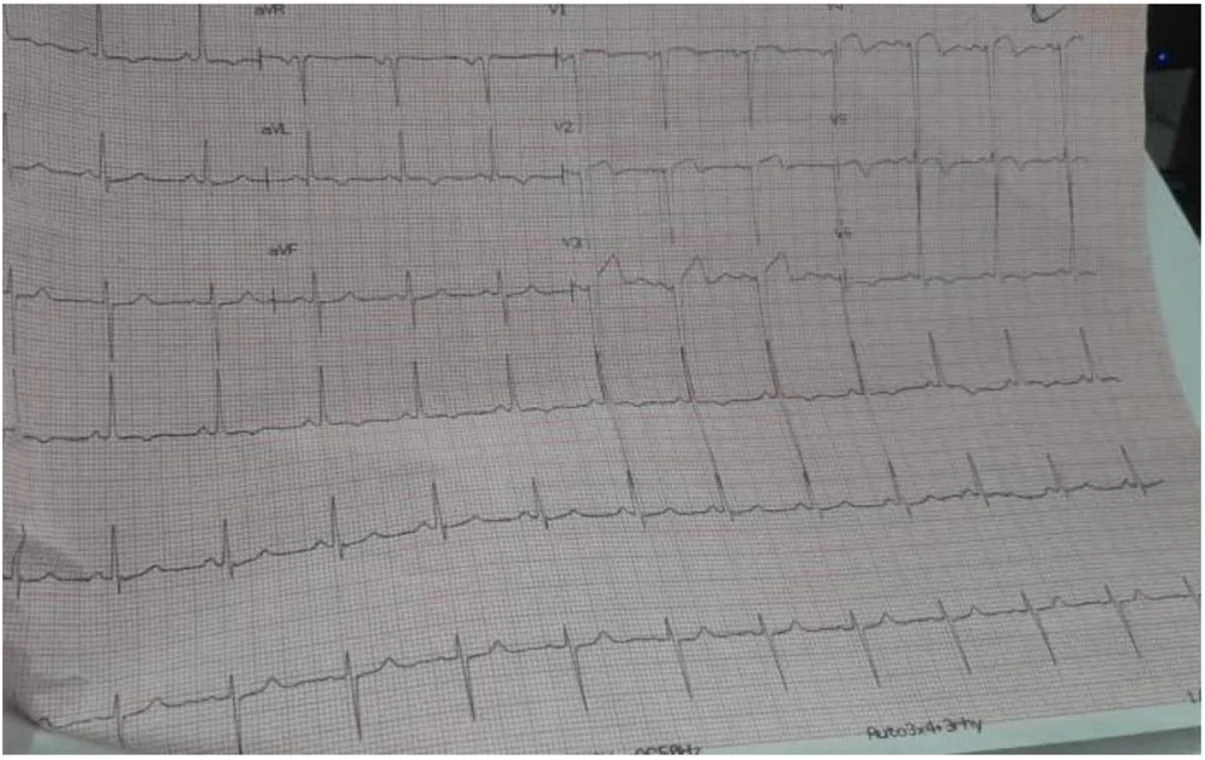
Digital ECG export on admission showing ST-segment elevation in leads V1–V4 at the peak of elevation.

**Figure 2.**
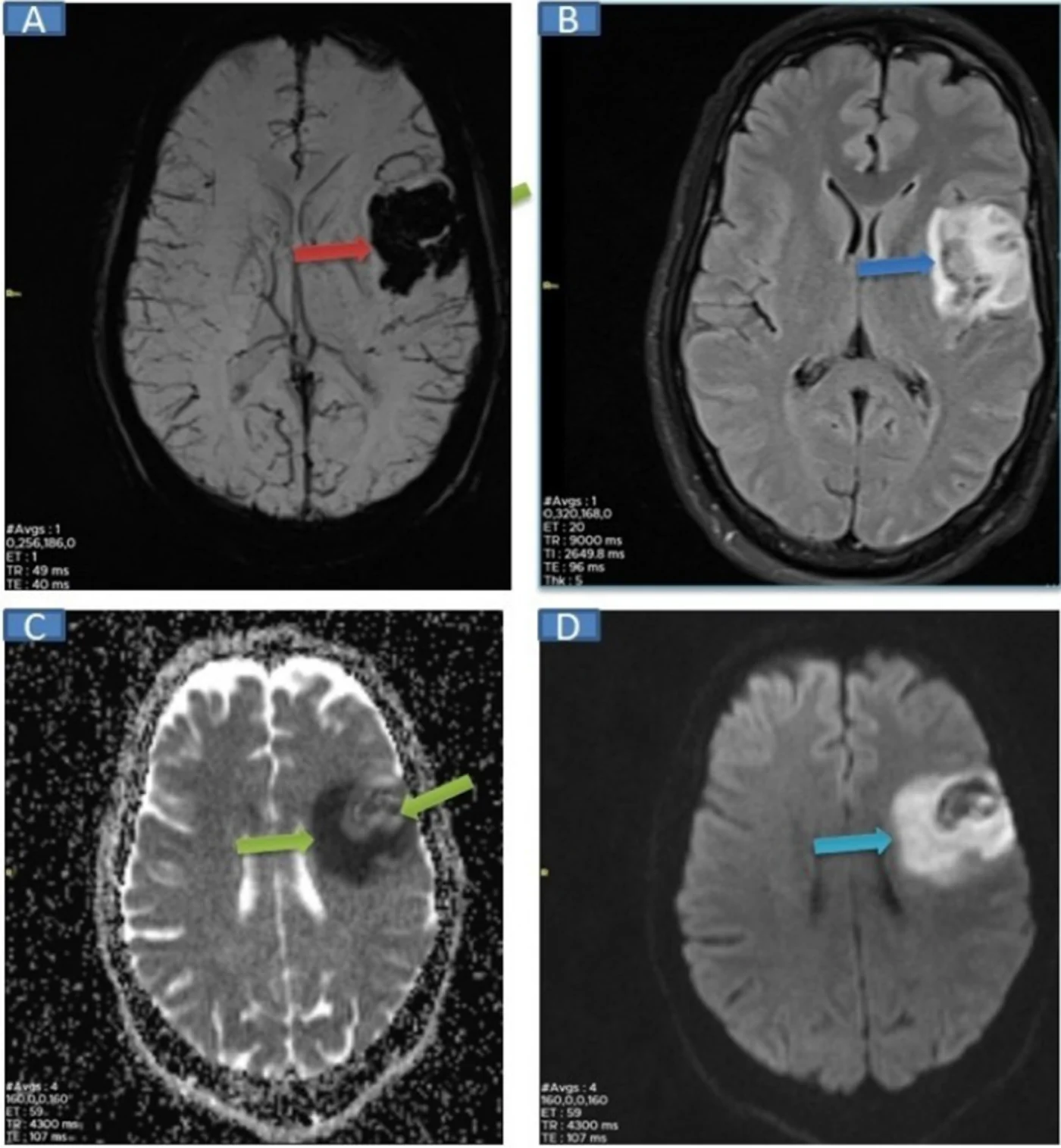
Imaging of left parietotemporal hemorrhagic ischemia. SWI shows blooming artifacts (red arrow), FLAIR demonstrates hyperintense signal (blue arrow), DWI reveals high signal intensity (green arrow), and the ADC map shows a corresponding low signal (**light blue arrow**).

- ECG: ST-segment elevation in the anteroseptal leads (V1–V4; Fig. 1).
- Brain MRI: left parietotemporal infarction involving the insular cortex with hemorrhagic transformation, with DWI hyperintensity, low ADC values, and SWI blooming artifacts (Fig. 2).
- Echocardiography: preserved left ventricular ejection fraction (LVEF) (60–65%), mild LVH, and no regional wall-motion abnormalities. Takotsubo cardiomyopathy was considered and excluded.
- Laboratory: Serial troponin I: Day 1, 0.20 ng/mL; day 2, 0.15 ng/mL; day 3, 0.12 ng/mL; day 4, 0.10 ng/mL; and day 5, 0.08 ng/mL. BNP was 215 pg/mL on admission. Complete blood count and thyroid-stimulating hormone were within normal limits.
- Chest radiography: no acute cardiopulmonary abnormality.

HIGHLIGHTS

- ST-segment elevation can occur in acute ischemic stroke with hemorrhagic transformation and may mimic acute myocardial infarction.
- Neurogenic myocardial injury due to autonomic dysregulation is a key underlying mechanism.
- Involvement of the insular cortex plays a central role in brain–heart interaction, contributing to transient cardiac abnormalities.
- ECG abnormalities in neurogenic myocardial injury are often transient and resolve without the need for coronary intervention.
- Accurate differentiation from ST-elevation myocardial infarction is critical to avoid unnecessary invasive procedures and harmful antithrombotic therapy.
- Serial ECG monitoring, bedside echocardiography, and cardiac biomarkers are essential for proper management and patient safety.
- Conservative management with multidisciplinary coordination between neurology and cardiology ensures favorable neurological and cardiac outcomes.

### Diagnostic reasoning

The main challenge was differentiating acute coronary occlusion from neurogenic myocardial injury. Despite anteroseptal ST-segment elevation and elevated troponin, the preserved cardiac function, absence of regional wall-motion abnormalities, and primary neurological presentation supported a diagnosis of neurogenic myocardial injury. The differential diagnosis included acute STEMI, stress cardiomyopathy, and type 2 MI secondary to neurological stress and hypertension.

Although acute STEMI was initially considered because of ST-segment elevation in leads V1–V4 and mild troponin elevation, several clinical features argued against acute coronary occlusion as the primary diagnosis. The patient had no chest pain, no prior cardiac history, and presented primarily with acute focal neurological deficits. Echocardiography showed preserved left ventricular systolic function with an LVEF of 60–65% and no regional wall motion abnormalities. Serial troponin values demonstrated a mild downward trend rather than a progressive rise, and the ST-segment elevation resolved spontaneously by day 5 with conservative management. In the context of an acute left parietotemporal ischemic stroke involving the insular region with hemorrhagic transformation, these findings supported neurogenic myocardial injury as the most likely diagnosis. Nevertheless, because coronary angiography was not performed, occult coronary artery disease cannot be completely excluded.

### Management

**Figure 3.**
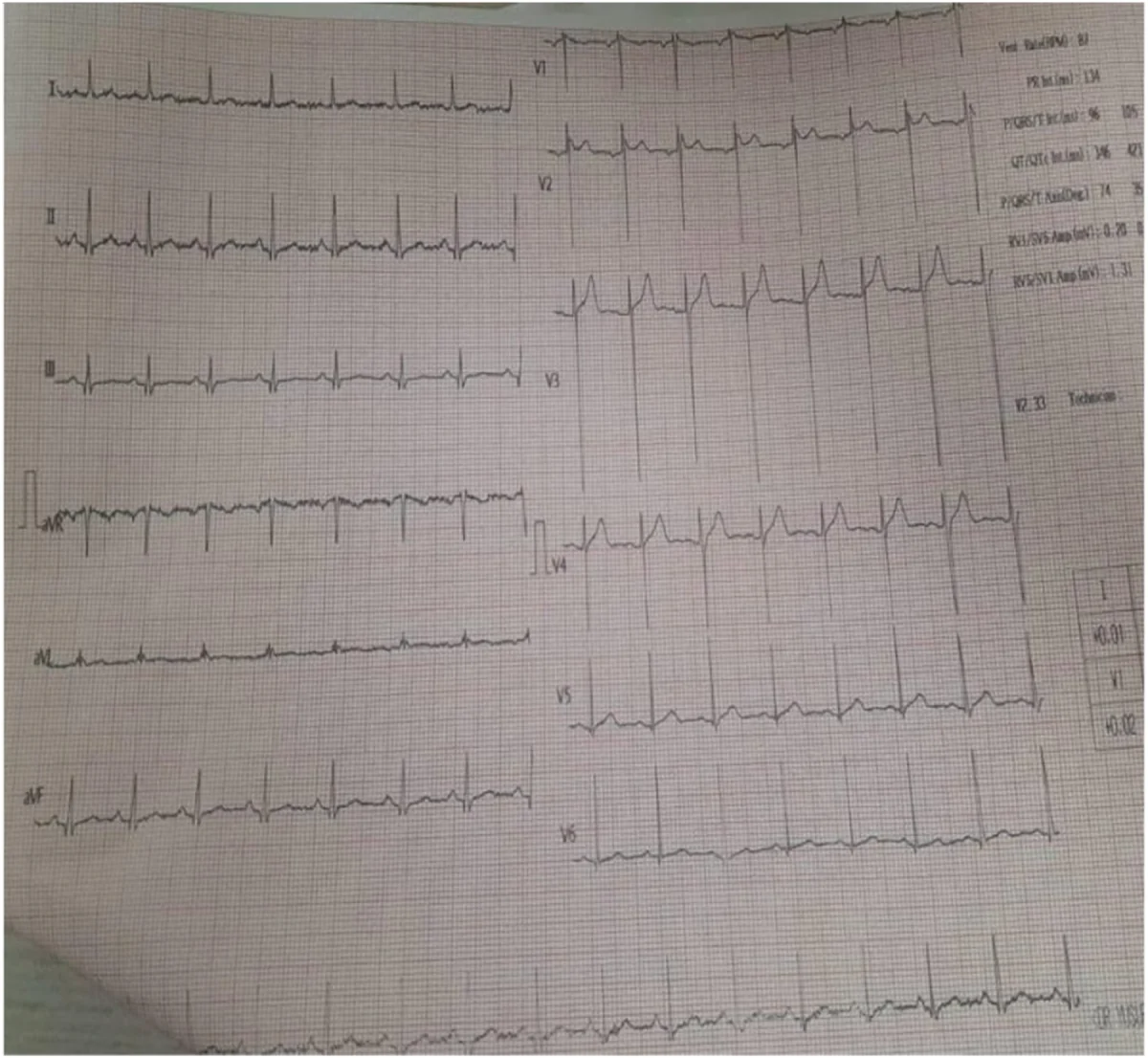
Follow-up digital ECG on day 5 showing normalization of ST-segment elevation compared with the initial peak.

The patient received intravenous antihypertensive therapy, mannitol infusion, head-of-bed elevation, supportive care, and inpatient physiotherapy. Serial ECG monitoring demonstrated resolution of ST-segment elevation by day 5 (Fig. 3). The patient showed progressive neurological improvement and was discharged with outpatient follow-up and continued rehabilitation.

## Discussion

Neurogenic ST-segment elevation following acute ischemic stroke with hemorrhagic transformation poses a significant diagnostic challenge due to its similarity to acute STEMI. Unlike primary coronary occlusion, neurogenic ECG changes are often transient, associated with neurological symptoms rather than chest pain, and typically lack mirror-image changes or persistent regional wall motion abnormalities^[^3,4^]^. Recognition of these differences is essential to prevent unnecessary invasive cardiac interventions and potential worsening of intracranial bleeding.

The pathophysiology involves the brain–heart axis, where acute neurological injury, particularly involving the insular cortex, triggers sympathetic overactivation and a catecholamine surge. Hemorrhagic transformation can amplify neurocardiac stress, causing myocardial stunning and transient ventricular dysfunction. These mechanisms explain the ST-segment elevation and elevated cardiac biomarkers observed in the patient, supporting a neurogenic rather than primary cardiac origin^[^3,4,6^]^.

The differential diagnosis includes acute STEMI, stress-induced cardiomyopathy, and type 2 myocardial injury secondary to neurologic stress and hypertension. The absence of clinical instability, preserved left ventricular function, and spontaneous resolution of ECG changes favor neurogenic myocardial injury^[^5,6^]^.

The presence of hemorrhagic transformation was central to management decision-making. In a patient with intracranial bleeding, empiric antithrombotic therapy or urgent invasive coronary intervention may increase the risk of hematoma expansion or neurological deterioration. Therefore, the management strategy required balancing the possibility of acute coronary syndrome against the immediate neurological bleeding risk. In this case, the absence of chest pain, preserved systolic function, lack of regional wall motion abnormality, stable hemodynamics, and spontaneous ECG improvement supported close cardiac monitoring rather than immediate invasive coronary evaluation.

A limitation of this case is the absence of coronary angiography, which means that occult coronary artery disease cannot be fully excluded. Despite this, the clinical course and imaging support a neurogenic mechanism^[^5,6^]^.

Management should be conservative, with close monitoring of vital signs, serial ECGs, and cardiac biomarkers. Bedside echocardiography is recommended to assess ventricular function dynamically. Multidisciplinary coordination between neurology and cardiology is critical to optimize patient outcomes and prevent iatrogenic harm^[^4,5,8^]^.

Finally, the correlation between the normalization of ECG findings and progressive neurological recovery reinforces the transient, reversible nature of neurogenic myocardial injury in the setting of an acute ischemic stroke with hemorrhagic transformation.

### Limitations

A key limitation of this case is the absence of coronary angiography or coronary CT angiography. Therefore, acute coronary syndrome and occult coronary artery disease cannot be definitively excluded. However, the clinical presentation, preserved echocardiographic function, absence of regional wall motion abnormalities, mild and declining troponin elevation, and complete resolution of ST-segment elevation with conservative management make acute coronary occlusion less likely. This case should therefore be interpreted as probable neurogenic ST-segment elevation rather than as angiographically proven exclusion of coronary artery disease.

## Conclusion

Neurogenic ST-segment elevation can occur in patients with acute ischemic stroke complicated by hemorrhagic transformation and may closely mimic acute STEMI. Accurate diagnosis relies on correlating neurological findings, ECG changes, cardiac biomarkers, and echocardiographic assessment. Recognition of neurogenic myocardial injury is crucial to avoid unnecessary invasive cardiac procedures and antithrombotic therapy, which may exacerbate intracranial bleeding. Conservative management with serial ECG monitoring, bedside echocardiography, and dynamic biomarker evaluation is recommended. Multidisciplinary collaboration between neurology and cardiology teams ensures patient safety and optimal outcomes. Early identification and careful monitoring can result in complete resolution of ECG changes and favorable neurological recovery.

Artificial intelligence-assisted language editing was used during manuscript preparation to improve grammar, clarity, formatting, and alignment with the SCARE 2025 checklist. ChatGPT, developed by OpenAI, was used through a cloud-based interface in April 2026. Only de-identified manuscript text was provided. No patient identifiers, clinical decision-making, image generation, statistical analysis, or diagnostic interpretation were performed by AI. All AI-assisted content was reviewed, edited, and verified by the authors, who take full responsibility for the final manuscript^[^9^]^.

## Data Availability

The data supporting the findings of this study are available from the corresponding author upon reasonable request.
